# Real-Time Stress Assessment Using Sliding Window Based Convolutional Neural Network

**DOI:** 10.3390/s20164400

**Published:** 2020-08-07

**Authors:** Syed Faraz Naqvi, Syed Saad Azhar Ali, Norashikin Yahya, Mohd Azhar Yasin, Yasir Hafeez, Ahmad Rauf Subhani, Syed Hasan Adil, Ubaid M Al Saggaf, Muhammad Moinuddin

**Affiliations:** 1 Center for Intelligent Signal and Imaging Research (CISIR), Electrical and Electronics Engineering Department, Universiti Teknologi PETRONAS, Bandar Seri Iskandar 32610, Malaysia; syed.farazn.is@gmail.com (S.F.N.); norashikin_yahya@utp.edu.my (N.Y.); yasir_hafeez@ymail.com (Y.H.); raufsubhani@gmail.com (A.R.S.); 2 Department of Psychiatry, Universiti Sains Malaysia Health Campus, Kota Bharu 16150, Malaysia; mdazhar@usm.my; 3 Department of Computer Science, Iqra University, Karachi 75500, Pakistan; hasan.adil@iqra.edu.pk; 4 Center of Excellence in Intelligent Engineering Systems, Department of Electrical and Computer Engineering, King Abdulaziz University, Jeddah 21589, Saudia Arabia; usaggaf@kau.edu.sa (U.MA.S.); mmsansari@kau.edu.sa (M.M.)

**Keywords:** stress-assessment, CAD (computer-aided diagnosis), machine learning, convolutional neural network, feature extraction, real time, sliding window

## Abstract

Mental stress has been identified as a significant cause of several bodily disorders, such as depression, hypertension, neural and cardiovascular abnormalities. Conventional stress assessment methods are highly subjective and tedious and tend to lack accuracy. Machine-learning (ML)-based computer-aided diagnosis systems can be used to assess the mental state with reasonable accuracy, but they require offline processing and feature extraction, rendering them unsuitable for real-time applications. This paper presents a real-time mental stress assessment approach based on convolutional neural networks (CNNs). The CNN-based approach afforded real-time mental stress assessment with an accuracy as high as 96%, the sensitivity of 95%, and specificity of 97%. The proposed approach is compared with state-of-the-art ML techniques in terms of accuracy, time utilisation, and quality of features.

## 1. Introduction

Mental stress is a state of mind in which the brain and body respond to pressures from a situation or life event which could be due to internal, external, physical, psychological, or social factors [1]. The coping mechanism of the brain is triggered [2] during which the brain decelerates normal body functions and prioritise blood flow and rapid breathing in an effort to keep the mind and body alert. Recurring stressful conditions can render a person susceptible to other mental disorders, i.e., depression, anxiety, or emotion disorders [3]. Furthermore, stress can increase the risk of heart attack, cardiac arrest, high blood pressure, etc. [4]. It can affect cognitive functions and interfere with memory, resulting in memory weakness [5]. Habib et al. [6] presented an extensive study regarding the effects of stress conditions over different functions of the body.

Diagnosing mental stress at the early stage is crucial to avoid further effects such as body dysfunctions. Current clinical practices involve questionnaire-based evaluations, such as perceived stress scale [7], stress response inventory [8], and life events and coping inventory [9], which can assess stress conditions. These evaluations are highly subjective and do not reflect the current state of mind in most cases. Furthermore, these questionnaires can be easily affected by the participants who are in denial [9]. Additionally, lack of awareness and denial regarding stress symptoms complicate stress diagnostics [10]. Therefore, to obtain a more reliable evaluation, several computer-aided diagnosis (CAD) systems have been employed based on different modalities such as electrocardiography (ECG), skin conductance, facial expression, blood pressure, functional near-infrared spectroscopy (fNIR), and electroencephalography (EEG).

EEG is a non-invasive and low-cost technique that can provide high temporal brain activity measurements, rendering it highly suitable for many clinical environments for brain assessments. However, interpreting an EEG signal requires a reasonable amount of time and expertise for diagnosis [10] and is prone to errors [11]. A misdiagnosis may cause a non-stressed person to be identified as a stressed one and vice versa. This may result in either treatment withdrawal or further worsening of the participant’s mental condition. J. Kaye et al. [12] discussed possible links between stress and depression and reported that stress could result in clinical depression. Similarly, conditions such as unattended post-traumatic stress disorder (PTSD) with depression may induce suicidal behaviours [13,14]. CAD evaluation of EEG signals has been performed using machine learning (ML) to identify EEG patterns for predictions involving PTSD or depression [15,16,17,18]. Furthermore, using CNNs, deep learning has been applied to classify depression-related EEG signals [16].

CAD techniques using ML approaches for stress assessment have been reported [16,19,20,21]. Most of these systems are either offline or time-consuming methods. Vanitha et al. [22] reported a real-time stress detection system. However, they have not considered the required response time for real-time systems. Jiayuan He et al. [23] compared the use of conventional machine learning approach with CNN for real-time stress assessment with the help of ECG signals. The study uses 10 s of ECG signals. For a real-time CAD system to perform well, all processing must be completed within the allowable time window for decision making. CAD systems that rely on ML approaches require pre-processing and supervised feature extraction; these processes are inefficient, tedious, and sensitive to errors. Hence, the performance of CAD systems is deteriorated in terms of accuracy, which renders them unsuitable for real-time applications, such as neurofeedback techniques that allow the subject to train the corresponding brain functions [24]. For example, a stress assessment application for wearable devices has been reported by J. Minguillon et al. [25]; however, this method depends on an offline process computation.

**Figure 1:**
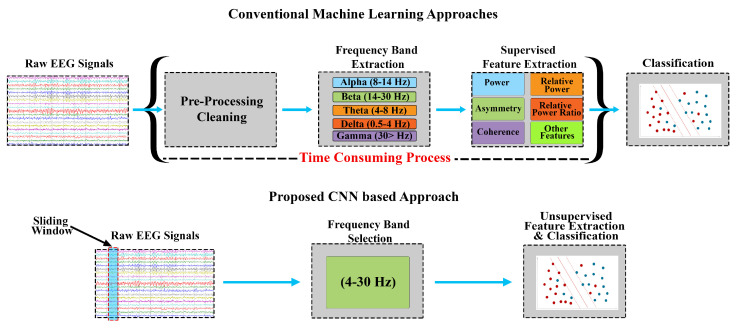
Comparison between proposed and general stress assessment techniques, the proposed approach eliminates the pre-processing cleaning, individual bands extraction and supervised feature extraction phases.

Recently, CNNs have been used in detecting fraudulent bank transactions from real-time streaming data [26]. CNN-based approaches have been proven to be more efficient compared with conventional ML approaches [27,28] in terms of signal size, label, and quality of features extracted. Therefore, owing to CNN’s generalised and automated unsupervised feature extraction, we propose using CNNs for stress assessment and detection. This approach is expected to avoiding tedious, time-consuming, and error-prone tasks of feature engineering involved in ML approaches. The intention of this study is to explore different techniques that can provide stress assessment in minimum time so that it can be compatible with real-time assessment, for this purpose many approaches can be used which have the capacity to avoid the time-consuming factor in a machine learning approach and still achieve higher accuracy. A comparison of the approaches is demonstrated in Figure 1, where the pre-processing and supervised feature extraction and selection steps required in conventional ML approaches have been avoided in the proposed CNN-based stress assessment approach. Hence, CNNs were used for real-time stress assessment using EEG signals in this study. The performance was compared in terms of accuracy, time utilisation, and quality of features. Section 2 details the proposed approach and description of the experiments, followed by results and analysis Section 3.

## 2. Proposed CNN Based Real-Time Stress Assessment

The proposed real-time stress assessment approach is based on employing a CNN that can handle a large amount of data and unsupervised feature extraction. The proposed approach, as shown in Figure 1, comprises of three main process blocks: data acquisition, frequency band selection, and classification. The proposed approach takes raw EEG data in real-time and does not require individual EEG band extraction other than a single 4–30 Hz band filtering. The features are extracted in an unsupervised manner from a sliding window.

The following subsections elaborate the proposed method and describe the data and experiments. The experiments were conducted to analyse and compare the proposed approach with conventional ML approaches. This includes the use of raw EEG data, suitability for real-time applications, data labels for stress assessment, and quality of unsupervised features.

### 2.1. General CNN Architecture

**Figure 2:**
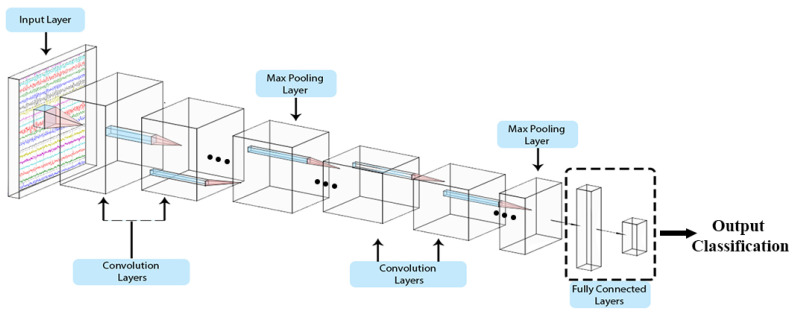
General CNN.

The ability of CNNs to extract spatial features to a granular level and generalised feature matrices provides a high discrimination power for classification. Typically, CNNs are used for image-related objectives; however, they can also be used on signals [29,30]. A CNN comprises primarily three layers: convolutional, pooling, and fully connected layers as shown in Figure 2. Each layer contains filter windows that slides over the input layer from the preceding layer. Filter size, strides (sliding of widows), and padding (window offset over input) settings are parameterised.

#### 2.1.1. Convolutional Layer

The convolution layer contains kernel; these kernels slide over the input to perform initial unsupervised feature extraction. This phenomenon can be expressed as follows [31]:

(1) $$y_{i^{l+1},j^{l+1},d}=F*X$$

(2) $$F*X=\sum_{i=0}^{H}\sum_{j=0}^{W}\sum_{d^{l}=0}^{D^{l}}f_{i,j,d^{l},d}\times x_{i^{l+1}+i,j^{l+1}+j,d^{l}}^{l}$$

where the above Equation (1) is repeated for all 0≤d≤D=Dl+1, and for any spatial location $\left(i_{1}+1,j_{1}+1\right)$ satisfying $0\leq i_{1}+1<H_{1}-H+1=H_{1}+1,0\leq jl+1<Wl-W+1=Wl+1$. In this equation $x_{i^{l+1}+i,j^{l+1}+j,d^{l}}^{l}$ refers to the element of $x^{l}$ indexed by the triplets $i^{l+1}+i,j^{l+1}+j,d^{l}$. The convolution layer is followed by the activation function such that the model is more robust and sensitive toward noise in the signal. A leaky-relu is used as the activation function at layers four and ten, which, unlike the relu, allows a small nonzero gradient when the unit is inactive, as shown in Equation (3).

(3) $$f\left(x\right)=\left\{\begin{array}{c} x,\;\mathrm{if}\;x>0\\ 0.01x,\;\mathrm{if}\;x\leq 0 \end{array}\right.$$

#### 2.1.2. Pooling Layer

This layer reduces the feature map obtained from the convolution layer. Different techniques can be used in this regard; the most popular is the max pooling technique, which extracts the most significant feature within the defined region of a feature map.

#### 2.1.3. Fully Connected Layer

This layer can be an analogy of the fully-connected artificial neural network (ANN) because it comprises every neuron connected to every other neuron of the preceding layer. The features are flattened into one dimension before the fully connected layer. The following Equation (4) expresses the process.

(4) $$x_{i}=\sum_{j=0}^{N-1}w_{j}y_{j}+b_{i}$$

where *w*, *b*, *x*, and *y* represent the weights, biases, final output, and the output from the previous layer, respectively. The softmax function predicts based on *x* from Fully-connected layer for the class of patterns, i.e., stress and non-stress.

### 2.2. Data Structuring/ Transformation (CNN)

**Figure 3:**
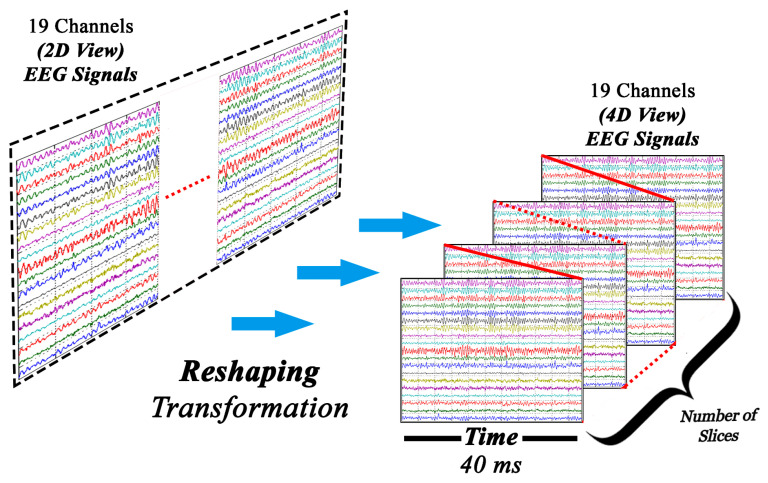
Transformation of 2D to 4D signal, the 4D transformed signal comprises of the signal values, channels, window length in time and number of slices.

Stress EEG patterns are instantaneous and last for short time intervals in real-time. Therefore, developing a real-time stress assessment approach requires to process and extract features from the instantaneous signal windows. This makes the batch processing of the entire EEG data for real-time stress assessment inefficient as the features representing stress patterns may be lost. In this paper, a window of 40ms is considered, based on the analysis for maximum feature retention (discussed in Section 2.7.3). In order to employ CNN, signals are segmented into 40 ms windows and eventually into a four-dimensional (4D) matrix in Matlab, as required by CNNs. Figure 3 shows the transformation of a two-dimensional (2D) signal into a 4D conceptual format, which is a prerequisite for CNNs. The signal is divided into 40ms windows, in which each window contains 19 electrode channels, 20 samples, and a depth of 1. Each signal slice is 40 ms apart.

### 2.3. Band Selection

EEG signals contain different frequencies; these frequencies refer to different functions of the brain at different locations. Based on brain activities, each band contains different ranges of these frequencies. The well-known frequency bands are alpha, beta, gamma, theta, and delta. In conventional machine learning approaches, each band is filtered individually from the signal and then feature extraction is applied over all bands, making it both time and computationally inefficient. In this study, the individual bands are not considered, rather the frequency band between 4–30 Hz is considered from the raw EEG signals. This is also justified as a number of researchers have reported that the stress patterns are in between 4–30 Hz frequency range [32,33,34]. Therefore, the time and computational complexity of the process is reduced as only one filter is required, whereas the conventional machine learning approaches require multiple filters for each frequency band.

### 2.4. CNN Architecture

To classify stress from EEG signals, spatial information is vital. In this study, we investigated the performance of the CNN architecture for stress assessment and classification. The architecture includes a four-layer-deep CNN model with two CNN layers connecting consecutively without an activation function between them. The activation function used in this CNN model was the leaky-relu to avoid the dead neurons during learning. The convolutional layers are followed by a single, fully connected layer, which will provide the outcome of the classification. Furthermore, 50% of the neurons were randomly turned off at each iteration to generalise the model, which was performed by adding a dropout layer, and the dataset was shuffled at each epoch to avoid overfitting. Subsequently, 32 batch sizes and 20 epochs were used for training.

The optimiser used for this training was the Adam optimiser, and the cross-entropy loss function was selected. The dimensions of the feature matrix were reduced using pooling but essential features were maintained using max-pooling after convolution. To avoid overfitting and underfitting in the model, the dataset was divided into training, validation, and testing data. The parameters were obtained after optimising the accuracy of the model, and the details of the model architecture are shown in Table 1.

### 2.5. Description of Dataset

In this research, we have utilized the dataset of Subhani et al. [35] who collected the data aiming to classify stress levels. The data acquisition setup comprised Net Amp 300 amplifier (Electrical Geodesic In. (EGI), USA) with 128 electrodes referenced to Cz. The impedance of all electrodes was kept below 50 KΩ. The signals were recorded at a sampling rate of 500 samples per second with a 50 Hz notch filter to protect signals from line noise. In the experimental setting, a computer-based mental arithmetic task induced stress conditions, which applied the Montreal imaging stress task (MIST) protocol [36], which has three sessions: relaxation, and four levels of each of stress and control. The experiment took place between 3 pm and 7 pm. The experiment designed is approved by the Ethics Commission at Hospital Universiti Sains Malaysia, Malaysia.

**Figure 4:**
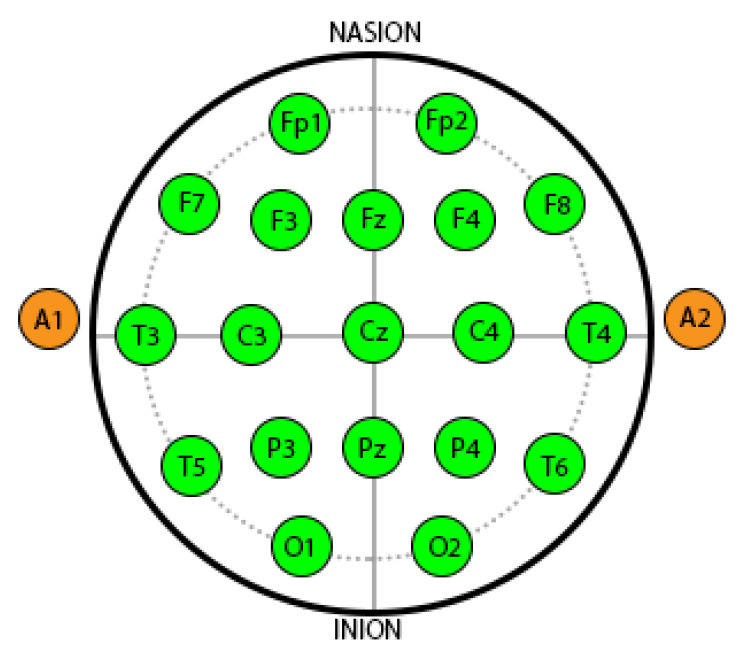
Locations of 10–20 electrodes.

In this study the data for 26 subjects (average age: 22.46 ± 0.79, six females, and four left handed) is used. The selected participants had no previous critical medical history and were free from any neurological abnormalities. They exhibited normal or corrected-to-normal vision. The participants were fasting for at least 2 h before starting the experiment. The EEG recording comprised of two sessions: relaxing and stress periods for 5 min each, which resulted in 260 min of EEG recording for the study resulting in 15,000 windows of 40ms duration for each subject. In total, 19 electrodes were used after selection based on 10-20 montage with an average mastoid reference (labelled as Fp1, Fp2, F3, F4, F7, F8, C3, C4, T3, T4, T5, T6, P3, P4, O1, O2, Fz, Cz, and Pz). Figure 4 shows the different locations of the electrodes.

**Figure 5:**
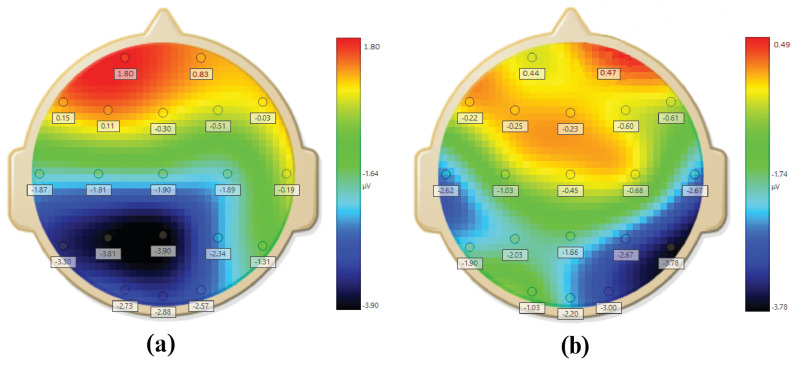
Average topomap images of brain for (**a**) stressed and (**b**) non-stressed subjects [41].

It is reported in the literature that negative emotions and withdrawal behaviours [37,38,39,40] affect the prefrontal alpha asymmetry. Several researchers have used AAS value for stress assessment [40,41,42,43]. In general, a low value of alpha asymmetry indicates that the participant is in stress [5,19]. Lehrer et al. [44] provided in-depth knowledge regarding the principles and practices for stress management. They also highlighted evidence regarding the effects of relaxation and stress over the alpha band. The characteristics of alpha-band and their correlation with stress and depression have been reported in several papers [45,46,47]. Therefore, AAS is used in this study to assign labels for stress assessment. The windows are labelled using AAS values between Fp1 and Fp2 on prefrontal region as shown in Figure 4. Topomaps for stressed and non stressed subjects based on AAS are shown in Figure 5.

### 2.6. Training, Validation, and Testing

The training phase of the model was performed using the hyperparameters as mentioned in Table 1 with the data structure and architecture discussed in the transformation details in Section 2.2 the transformation details. The data for 20 subjects were used for training with a total of 300,000 windows. The stress and non-stress patterns were labelled based on AAS values. Out of 300,000 (20 × 15,000) windows, a total of 45,000 windows were labelled as stressed based on AAS values. Therefore, 45,000 non-stressed windows were selected, making the entire size of the training dataset to be 90,000 windows. The validation was performed on the entire data for three subjects and the testing was performed on the remaining three subjects that were completely blind during the training and validation phase. The dataset was shuffled at every epoch to reduce the case of overfitting and to generalise the CNN model. The performance metrics were computed from the confusion matrix as follows:

(5) $$Sensitivity=\frac{TP}{TP+FN}$$

(6) $$Specificity=\frac{TN}{TN+FP}$$

(7) $$Accuracy=\frac{TP+TN}{TP+TN+FP+FN}$$

The sensitivity of a model represents its ability to identify correct positive cases and interpreted with the classification of true cases/positives, represented in Equation (5). Specificity corresponds to the ability of a model to identify non-cases correctly and is often denoted as true negatives, defined as shown in Equation (6). Conventionally, the confusion matrix represents the accuracy of the trained model (7), which is an average between the sensitivity and specificity.

### 2.7. Experiments and Bench-Marking Details

#### 2.7.1. Stress Assessment Based on ML

The work presented in [21] performs stress assessment using features extracted from EEG. Each EEG recording session of the participant contains 5 min of brain activities. Each session labelled as a stressful or nonstress session. The ML approach can learn different patterns that may be associated with the stress patterns; as such, it can be used to automate the analysis of stress patterns. The features extracted were power, relative power, relative power ratios, coherence, and asymmetries. The data were denoised, thereby removing artefacts that could directly affect the learning process of the machine before feature extraction. The algorithms suggested in this study are support vector machine (SVMs) with the radial basis function. The scope of the study was to only assess the stress level of the participants and does not involve any procedure by which this process can apply to real-time stress assessments. The experimental protocol is suitable for offline assessments because of the length of the signal data, which is 5 min of the EEG signals.

#### 2.7.2. Stress Assessment Based on ML with Raw EEG

To assess the technique presented in [21,35], which used clean data, the ML techniques were assessed with the same numbers of features and length of the signal but raw data that contained both noise and artefacts. The algorithms used in this case were the decision tree (DT), logistic regression (LR), and SVM, which are typically used for signal data classification. Hence, the effect of each process change is more visible over the classification ability by the standard algorithms.

#### 2.7.3. Conventional Batch-Wise vs. Window-Based Feature Extraction

**Figure 6:**
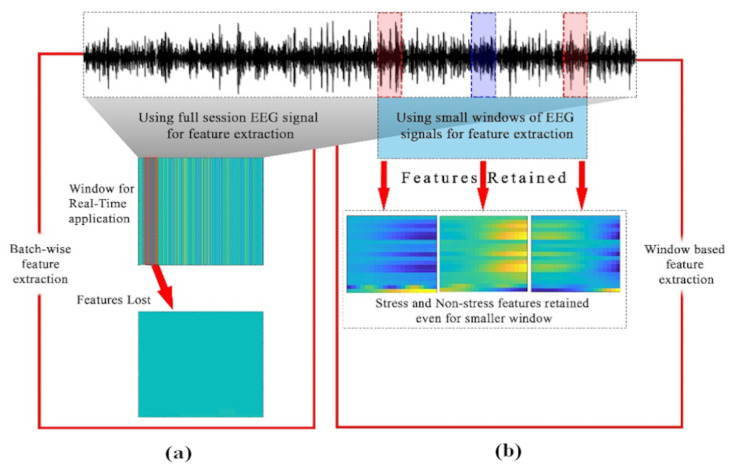
Feature Retention. (**a**)—features extracted from the whole EEG session will be lost if applied to 40ms window for real-time applications. (**b**)—features are retained when extracted from small sliding windows.

**Figure 7:**
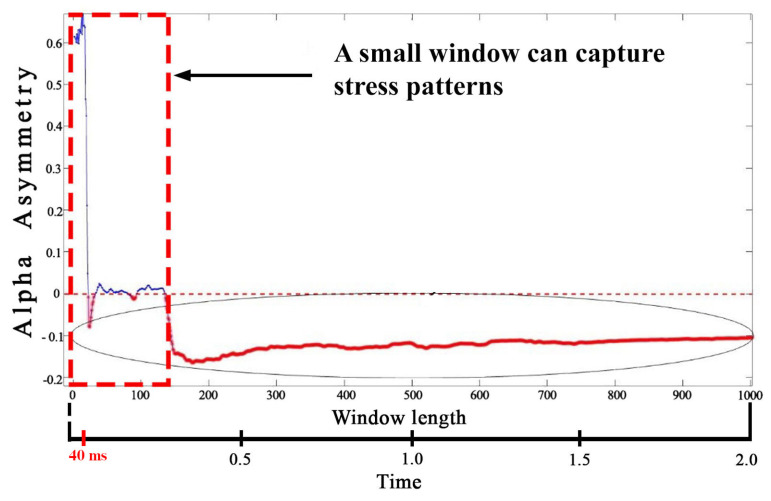
Effect of different signal lengths over AAS value; 40 ms windows are better in terms of capturing and classifying stress patterns based on AAS value.

For a real-time application, batch processing of the entire EEG signal data is not suitable. For example, neurofeedback applications require signal evaluation within less than 1 second for content triggering. Hence, feature extracted from the entire data may not represent the stress patterns contained in 40ms window. The advantage of using sliding windows over the batch signal is its compatibility with real-time assessments and prevention of feature loss. To demonstrate the prevention of feature loss, feature extraction is performed on 40ms windows as well as on the entire batch signal. Figure 6 shows that the 40 ms windows retained the features representing the stress patterns. Whereas, the features extracted from the batch data were not suitable while applying on 40ms window for real-time applications. For example one metric for stress assessment is AAS value between channel Fp1 and Fp2 at prefrontal region [5]. Figure 7 shows that the AAS values (used for labelling) may shift from negative to positive when the length of the signal window is incremented gradually. Moreover, the window size of 40ms results in an optimal value of AAS showing that the stress and non-stress patterns are not lost. This shows that the stress patterns can be shadowed by non-stress patterns if a longer window is considered.

#### 2.7.4. Stress Assessment Based on ML and CNN with AAS Labels

To assess the effect of the asymmetric analysis, each window was labelled with AAS. The stress condition was instantaneous because denoting an entire session with a single label was inefficient because many of the patterns would be overlapped with each other, implying that in a single session, both nonstress and stress conditions can appear, thereby affecting the ability of the model to classify signals. The experiment included the training of previous learning algorithms, i.e., SVM, LR, and DT with 40 ms windows of raw data; each window was labelled with a new AAS label. Feature extraction from these windows included the power, relative power, relative power ratios, coherence, and asymmetries (eliminating the AAS between prefrontal cortex FP1–FP2). The experiments relied upon the ML approach, which was dependent on the features extracted for the learning algorithm in a supervised manner. Hence, the ability of the ML algorithm depended on the extracted features. The classification between the classes would be inefficient if the features were inefficient. Hence, deep learning can be pivotal for replacing the dependency of classification over the supervised features. To avoid the process of supervised feature extraction and selection, the concepts of deep learning are useful. The CNN, a technique from the pool of deep learning approaches, can extract spatial features from raw signal windows in an unsupervised manner for classification. After training, the model is validated and tested over unseen data to evaluate its ability to perform efficiently with other blind data, which represents the generalisation capability of the model. Cross-validation includes the classification of features extracted by the CNN from ML algorithms to assess the efficiency of features extracted by the CNN.

## 3. Results

The proposed network was trained, validated, and tested with the proposed hyper-parameters presented in Table 1. The performance of the model was tested and measured by the metrics shown in Equations (5)–(7). The results obtained demonstrated the capability of the model in classifying correct stress and nonstress patterns. The model yielded 96% accuracy, 95% sensitivity, and 97% specificity, with raw data and compared with different techniques, i.e., LR, Gaussian SVM, and coarse tree on EEG signal batch-wise, process as detailed in [20,21,48]. In [21], features were extracted from processed EEG signals and classified using the SVM with the radial basis function, which yielded classification results with 79.54%, 78%, and 81% accuracy, sensitivity, and specificity, respectively in [21] whereas 94.6% accuracy is reported in [35] for 2 levels stress assessment.

However, the procedure reported in [21,35] is not suitable for real-time applications owing to the pre-processing of EEG data, large batch sizes of signals, and it included supervised features extraction, which is sensitive; these constraints render it difficult to address stress assessment for real-time applications. The scope of this study is to identify stress in real-time, for that reason in order to make a clear comparison among the existing techniques that address stress level identification from an offline EEG signals and the proposed method, we need to convert existing framework present in literature to make them compatible with the required process flow. The purpose of applying such conversion is the limitation that very few studies are available that address stress assessment in real-time with EEG signals.

### 3.1. Stress Assessment Based on ML with Raw EEG

**Figure 8:**
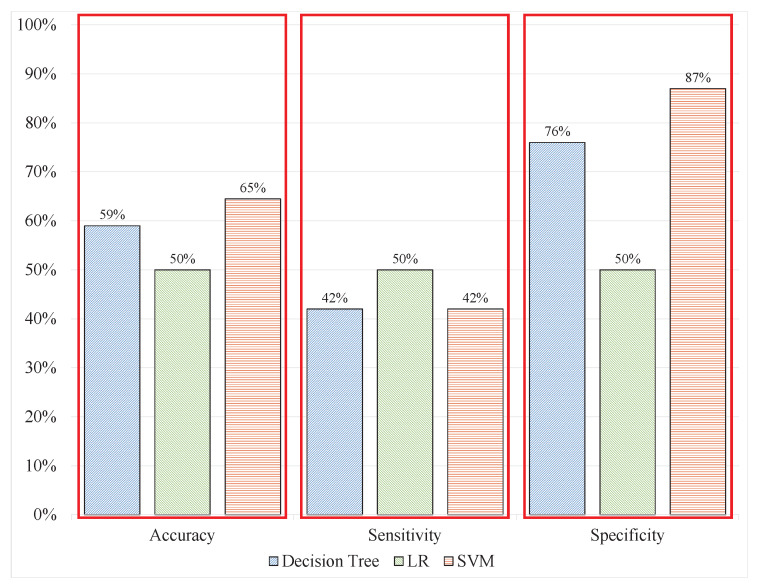
ML performance on raw EEG Signals in batch data.

To investigate the factors that can be improved to achieve a real-time assessment, the procedure was modified with different aspects to obtain the most optimised process. The EEG signals in [21] were processed using automatic cleaning techniques as well as manually removing noise and artefacts; therefore, they would not be compatible with real-time applications. In experiment 1, the same features from the same signal window size and labels were classified. The SVM, DT, and LR were used to classify the signal patterns. The DT produced classification results with 59.20%, 42%, and 76% accuracy, sensitivity, and specificity respectively; meanwhile, LR produced 50%, 50%, and 50% correspondingly, whereas SVM produced the most promising results in experiment 1 with 64.50%, 42%, and 87% accuracy, sensitivity, and specificity, respectively. Table 2 and Figure 8 demonstrate these results with a visualization showing that with uncleaned signal in batch data, the models could not perform correct classifications because the results deteriorated compared with the benchmark results from [21] that are 79.54% accuracy, 81% sensitivity and 78% specificity.

### 3.2. Stress Assessment Based on ML with Sliding Windows

**Figure 9:**
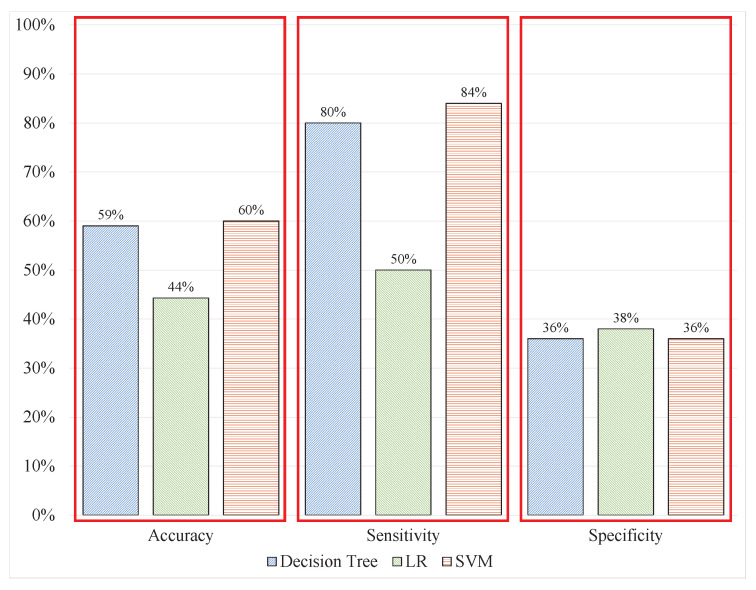
ML performance with sliding windows.

The use of uncleaned signals is inevitable when designing a system that avoided all manual processes for real-time applications. Hence, the signal window length can be changed to a 40 ms size. The feature extraction from sliding windows of signals includes the same features as in experiment 1 and the benchmark study with the same label associated with the batch window. After the extraction, the models were trained and tested over unseen signal data. The overall performance of the models deteriorated; the DT classified 58.8% of the signals correctly, whereas the LR could only identify 44.3% of the signal windows correctly, and the SVM predicted 60.7% of the signal windows correctly. The model’s performance demonstrated overfitting toward a single class because of the high sensitivity and low specificity of the models, as shown in Table 3 and Figure 9. This indicates that the performance of the models has slightly deteriorated from the previous experiment; the cleaning of EEG signals and feature extraction from batch data data increased the computational time of the entire process. Therefore, the system must address uncleaned signal data with a sliding window.

The labels provided were inefficient because the batch data may contain patterns belonging to both stress and nonstress conditions. Hence, the learned models could not identify the two classes correctly.

### 3.3. Stress Assessment Based on Ml with AAS Labels

**Figure 10:**
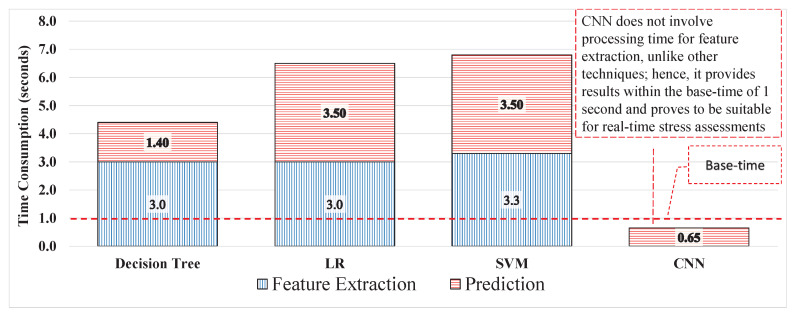
Performance comparison in terms of assessment time for real-time applications.

A new marker can resolve problems regarding labels. Each window was labelled using a new label and used for training a model. The performance of each model improved by 20–35%, the results from the DT improved from 58.8% to 84% with 91% sensitivity and 71% specificity compared with the previous experiment with 80% sensitivity and 36% specificity. LR could identify 84% of the signal windows correctly with 91% and 71% sensitivity and specificity, respectively. The SVM yielded results with 84%, 78%, and 90% accuracy, sensitivity, and specificity, respectively, which are shown in Table 4. Experiment 3 provided the best results when using AAS as a label with uncleaned 40 ms signals. However, to make the process compatible with real-time applications, it is essential to calculate the total time response of the process. Hence the computation of time response was for 1 second of the signals; the DT, LR, and SVM consumed 4.41, 6.87, and took 6.87 seconds to perform the prediction, respectively. The time response from all three techniques is not compatible for real-time applications because the system has to weigh for approximately 4–7 s to accept the new signal data. Prior to that, the data will increase with the multiple-frequency sample and time response. Hence, the time response for every second will increase exponentially, rendering the ML technique useless for real-time applications. The time responses of all techniques are compared in Table 4. As shown in Figure 10, the ML techniques consumed the most time during feature extraction based on the nature and quantity of the features, and different models have different time consumption abilities.

### 3.4. Stress Assessment Based on CNN with AAS Labels

The supervised feature extraction process must be replaced with other processes that are compatible with the real-time stress assessment system. The CNN in experiment 4 replaces the supervised feature extraction with an unsupervised extraction of features from the sliding windows with AAS labels. Throughout the experiments, the dataset was consistent; as such, the comparison should not be biased. After the training of the CNN model, it predicted the testing data, the same as in previous experiments. The classification performance of the CNN was 96% accuracy, 95% sensitivity, and 97% specificity with the time response for 1 s signal windows of 0.65 s. The model performed significantly better than all previous techniques discussed and performed all the processes in the minimum amount of time, thereby avoiding an exponential increase in the signal wait time, which occurred in the ML approach the performance is highlighted in Table 4 and the time response in Figure 10.

### 3.5. Cross-Validation of Features from CNN

**Figure 11:**
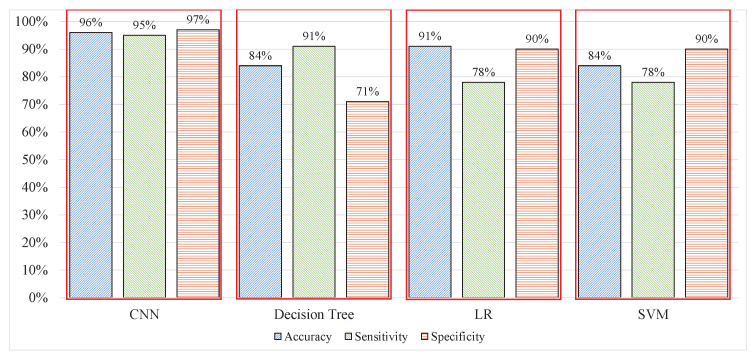
Performance comparison of CNN and ML techniques.

**Figure 12:**
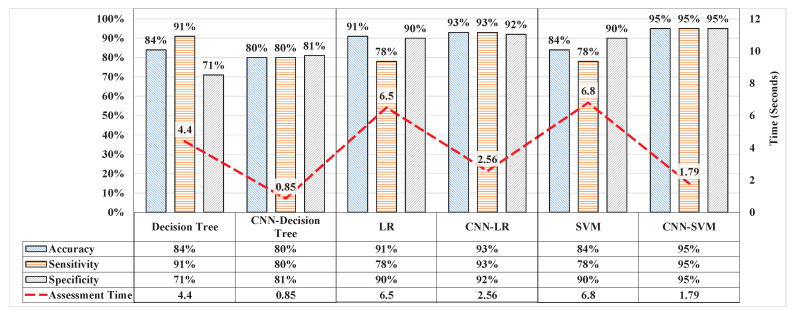
Performance of ML algorithms using supervised features as compared with features from CNN in terms of accuracy, sensitivity, specificity and assessment time.

The CNN must be validated to demonstrate that the features extracted are efficient. The features from the CNN were collected and used in the ML technique, i.e., DT, LR, and SVM. The classification results proved that the features from the CNN improved the performance capability of these ML techniques. The DT yielded 80.6%, 81%, and 80% accuracy, sensitivity, and specificity, respectively which shows decrement in case of accuracy as compared to the results produced by DT showed in Figure 11, but the model with supervised features is overfitting as the specificity is just 71% whereas with features from CNN made the model more robust. The performance of LR improved to 92.7% in terms of accuracy, with 93% sensitivity and 92.7% specificity. The SVM improved by 10% in terms of accuracy and yielded a total accuracy of 95% with sensitivity and specificity of 95% each, which was similar to the accuracy of the CNN, as demonstrated in Figure 12. The experimental results demonstrated that efficient features affected the performance of learning, and that the models performed well. When using ML techniques, domain experts performed feature extraction; however, after extraction, optimum features were selected during feature selection to ensure the good performance of the models.

The computation of the system as the time of model training depended on various factors that affected its operating time, i.e., the number of samples, number of epochs, and depth of the deep neural network. After model training, the minimum amount of time was required to process the data for classification. The signal filters consumed the most time in our process. The experiment results and comparisons were executed over MATLAB release version 2019 on DELL-OPTIPLEX 990 with hardware configuration of 8 GB RAM and Intel i7 3.4 GHz 2nd generation CPU.

## 4. Conclusions

This paper presents a real-time mental stress assessment method using sliding window based CNN. The study demonstrates the advantages of using CNNs over conventional ML techniques for real-time stress assessments on raw EEG data. The proposed approach yielded mental stress assessments in real-time and outperformed the conventional ML approaches in terms of accuracy, sensitivity, and specificity. The performance of the proposed approach was also analysed in terms of time utilisation, quality of features, and size of sliding window for real-time assessments applied to raw EEG data. The proposed approach can be utilised in real-time applications, such as neurofeedback training, and mobile and wearable stress assessment devices. Clinicians can also utilize the proposed approach to validate their psychiatric evaluations.

## Figures and Tables

**Table 1:** CNN architecture.

No.	Layer Name	Layer Parameter
1	Block Input	19 × 20 × 1 Images with zerocenter normalisation
2	Conv2D	16 3 × 3 convolutions with stride [1 1]
3	Conv2D	16 3 × 3 convolutions with stride [1 1]
4	MaxPooling2D	2 × 2 max pooling, stride = [1 1]
5	Leaky Relu	Leaky ReLu with scale 0.01
6	Dropout	50% dropout
7	Conv2D	10 2 × 2 convolutions, stride = [1 1]
8	Conv2D	10 2 × 2 convolutions with stride [1 1]
9	MaxPooling2D	2 × 2 max pooling with stride [1 1]
10	Leaky ReLu	Leaky ReLu with scale 0.01
11	Dropout	50% dropout
12	FC Layer	Two fully connected Layer

**Table 2:** ML performance on raw EEG Signals in batch data.

Performance/Techniques	D-Tree	LR	SVM
Data	Raw	Raw	Raw
Accuracy	59.20%	50%	64.50%
Sensitivity	42%	50%	42%
Specificity	76%	50%	87%

**Table 3:** ML performance with sliding windows.

Performance/Techniques	D-Tree	LR	SVM
Data	Raw	Raw	Raw
Accuracy	58.8%	44.3%	60.7%
Sensitivity	80%	50%	84%
Sepeciicity	36%	38%	36%

**Table 4:** ML techniques and CNN.

Performance/Techniques	CNN	DT	LR	SVM
Accuracy	96%	84%	84%	84%
Sensitivity	95%	91%	78%	78%
Specificity	97%	71%	90%	90%
Time consumption (1 s signal)	0.65 s	4.41 s	6.87 s	6.87 s
